# Editorial: Function of Renal Sympathetic Nerves

**DOI:** 10.3389/fphys.2017.00642

**Published:** 2017-08-28

**Authors:** Yutang Wang, Kyungjoon Lim, Kate M. Denton

**Affiliations:** ^1^School of Applied and Biomedical Science, Federation University Australia Ballarat, VIC, Australia; ^2^Neurophysiology, Department of Physiology, Anatomy and Microbiology, La Trobe University Melbourne, VIC, Australia; ^3^Cardiovascular Disease Program, Department of Physiology, School of Biomedical Sciences, Monash Biomedicine Discovery Institute, Monash University Clayton, VIC, Australia

**Keywords:** atherosclerosis, heart failure, hypertension, kidney disease, renal denervation, renal sympathetic nerves

## Introduction

Cardiovascular disease (CVD) is the leading cause of mortality and morbidity worldwide. The incidence of CVD is increasing in association with the growing prevalence of hypertension, diabetes, and obesity. This is occurring despite established effective therapies for hypertension. Thus, new methods for risk reduction are still needed (Cutler et al., 2008). The aim of this research topic was to evaluate the efficacy and safety of renal denervation (RDN), to explore the contribution of both afferent and efferent renal nerve activity to hypertension and non-hypertension disorders, and to stimulate future research to better understand the function of the renal nerves and the effects of RDN by highlighting gaps in knowledge.

## Evaluation of catheter-based renal denervation for the treatment of hypertension

Strong evidence that sympathetic overactivity is associated with the development of hypertension, combined with the demonstration that RDN prevents or delays hypertension in a variety of animal models, laid the groundwork for the introduction of RDN as a clinical therapy in humans (DiBona and Esler, 2010). In 2007, a novel, minimally invasive RDN ablation catheter was first trialed in hypertensive patients, with a 93% success rate of lowering blood pressure (BP) for at least 3 years post-RDN (Krum et al., 2014). These studies were met with acclaim (Lakhanpal and Domanski, 2012). However, a large scale, sham-controlled clinical trial (Symplicity HTN-3) failed to show reductions in BP greater than sham (Bhatt et al., 2014). It is now clear that poor trial design contributed to this outcome; only 19 of the 340 patients received at least the recommended 4+ ablations per artery and in these patients the falls in BP were greatest (Kandzari et al., 2015). In addition, the report by the Global SYMPLICITY Registry of 1,000 consecutively enrolled patients not only confirmed the safety of RDN but also suggested that RDN lowers office and ambulatory BP at 6 months post-procedure (Bohm et al., 2015). In the current volume, Fadl Elmula et al. summarizes the results of recent clinical RDN trials and rightly raised concerns regarding the interpretation of this data and called for caution in the implementation of this procedure. The authors point out that these trials did not confirm the marked BP lowering effect observed by initial RDN studies (Krum et al., 2009; Esler et al., 2010). In addition, they suggested that the BP lowering effect of RDN may be limited to discrete patient populations, and that it would be worthwhile searching for potential predictors of true responders to RDN. Briasoulis and Bakris reviewed the role of renal sympathetic nerves in hypertension and clinical applications of RDN in resistant hypertension. In light of the results from the Symplicity HTN-3 trial, the authors suggested that future randomized trials should be performed in experienced centers, using newer catheters, and in carefully selected compliant patients with true resistant hypertension.

## Effects of renal denervation beyond blood pressure control

It is noteworthy that RDN has also been shown to be effective in the treatment of other conditions coexisting with resistant hypertension. Thorp and Schlaich contributed a comprehensive review on RDN for hypertension and beyond. The non-hypertensive disorders that may also be affected by RDN include chronic kidney disease, chronic heart failure, left ventricular hypertrophy, arrhythmias, and metabolic diseases. Evidence suggests that clinical benefits beyond BP reduction could be gained following RDN in patients with these disorders. However, randomized controlled trials are needed before the application of RDN becomes routine in clinical practice for these patient cohorts. Moreover, these intriguing findings suggest that there is still a lot we don't understand about the function of the renal nerves.

### Heart failure

The kidney has a rich afferent sensory and efferent sympathetic innervation. It is now apparent that afferent renal nerve activity can reflexly modulate sympathetic outflow, not just to the kidney, but also to other organs, which may be one mechanism whereby RDN might improve outcomes in heart failure. Ramchandra and Barrett reviewed the mechanisms that contribute to enhanced renal sympathetic nerve activity (SNA) during heart failure, with an emphasis on afferent reflexes and central mechanisms. Booth et al. also contributed a review on this topic, focusing on critically assessing recent preclinical and clinical evidence supporting RDN as a potential treatment for heart failure. It was concluded that a reflex reduction in cardiac SNA in heart failure may be one beneficial effect of RDN. Booth et al. also explored the effect of RDN on the renal afferents, and provided evidence for both the destruction and then significant regrowth of afferent renal nerves. However, what effect ablating the afferent nerves play in mediating the responses to RDN is still largely unknown. Finally, Schiller et al. (2015) reviewed the contribution of renal nerves to the pathogenesis of chronic heart failure, providing an overview of clinical RDN studies to date. This culminated in a call for more research to determine how RDN may be employed as a safe and effective treatment in chronic heart failure.

### Kidney disease

Strong evidence demonstrates that renal SNA is elevated in both acute and chronic kidney disease. Although less robust, evidence also implicates increased afferent sensory nerve activity in kidney disease. In a primary study, Salman et al. demonstrated using telemetry-based recordings in rats with chronic kidney disease (Lewis Polycystic Kidney rats) that renal SNA directly correlated with BP. This study confirmed the notion that elevated renal SNA is likely a key contributor to hypertensive kidney disease. In an acute study, Goulding et al. examined the contribution of the renal nerves to baroreflex control of renal SNA and excretory function in cisplatin-induced renal failure in rats. The authors found that impaired renal function was associated with blunting of baroreflex gain and increased renal SNA, and that RDN restored these abnormalities. The results of this study suggested that dysregulation of renal SNA plays an important role in renal failure and that RDN may provide benefits for patients with kidney disease. In combination, this evidence led to the suggestion that kidney disease may also be amenable to RDN and Sanders and Blankestijn contributed a review of the clinical evidence arguing that RDN may have a place in the management of chronic kidney disease. RDN has been trialed in models of chronic kidney disease and been shown to be effective in lowering BP, though concerns were raised regarding the ability to adequately mount a response to hemorrhage (Singh et al., 2017).

## Long-term safety concerns following RDN

The introduction of catheter-based RDN initially moved forward at a rapid pace and though this has slowed, interest still remains strong. In part, this reduced momentum stems from concerns surrounding the lack of understanding of the long-term impact of RDN. Wang pointed out that although RDN is generally regarded as a safe procedure, some reports with small sample size have shown that the incidence of renal artery stenosis after RDN could be up to 18.2%; though this may depend on operator experience and the type of catheter employed. However, the safety of RDN needs to be continuously monitored.

In another study, Wang et al. examined the possible side effects of RDN in apolipoprotein E-deficient mice with hypertension induced by angiotensin II infusion. It was found that RDN increased atherosclerosis in the aortic arch despite a reduction in systolic BP in the mice. In contrast, RDN has been reported to decrease atherosclerosis in normotensive apolipoprotein E-deficient mice fed a high fat diet (Wang et al., 2015). Therefore, whether RDN promotes atherosclerosis in patients with resistant hypertension requires investigation in follow-up studies. In fact, the consequences of RDN in all patient populations in which this procedure is being trialed requires longer term follow-up, given evidence that the renal nerves regrow over time (Booth et al., 2015).

## Conclusion

Thus, the articles contained in this collection, contribute to our understanding of the function of the renal nerves, the effects of RDN as a treatment for hypertension and non-hypertensive disorders, and the side effects of RDN. However, much remains to be learned in terms of intra-procedural verification of the completeness of RDN, patient selection and long-term safety of RDN.

## Author contributions

YW, KL, and KD conceived the content. YW drafted the manuscript. KL and KD revised the manuscript.

### Conflict of interest statement

The authors declare that the research was conducted in the absence of any commercial or financial relationships that could be construed as a potential conflict of interest.
